# A survey of footwear advice, beliefs and wear habits in people with knee osteoarthritis

**DOI:** 10.1186/s13047-014-0043-8

**Published:** 2014-10-23

**Authors:** Kade L Paterson, Tim V Wrigley, Kim L Bennell, Rana S Hinman

**Affiliations:** Centre for Health, Exercise and Sports Medicine, Department of Physiotherapy, School of Health Sciences, Faculty of Medicine Dentistry & Health Sciences, The University of Melbourne, Melbourne, VIC Australia

**Keywords:** Knee, Arthritis, OA, Footwear, Shoes, Survey

## Abstract

**Background:**

Expert opinion recommends cushioned and supportive footwear for people with knee osteoarthritis (OA). However, little is known about the footwear advice people receive from healthcare professionals, or the beliefs and footwear habits of people with knee OA. This study aimed to determine i) what types of shoes people are advised to wear for their knee OA and by whom; ii) establish which types of shoes people with knee OA believe are best for managing their knee OA symptoms and (iii) which shoes they wear most often.

**Methods:**

204 people with symptomatic knee OA completed an online survey. The survey comprised 14 questions asking what footwear advice people had received for their knee OA and who they received it from, individual beliefs about optimal footwear styles for their knee OA symptoms and the types of footwear usually worn.

**Results:**

Only one third (n = 69, 34%) of participants reported receiving footwear advice for their knee OA, and this was most frequently received from a podiatrist (n = 47, 68%). The most common advice was to wear sturdy/supportive shoes (n = 96, 47%) or shoes with arch supports (n = 84, 41%). These were also amongst the shoe styles that participants believed were best for their knee OA (n = 157 (77%) and n = 138 (68%) respectively). The type of shoes most frequently worn were athletic (n = 131, 64%) and sturdy/supportive shoes (n = 116, 57%).

**Conclusions:**

Most people with knee OA who completed our survey had not received advice about footwear for their knee OA symptoms. Our participants typically believed that sturdy/supportive shoes were best for their knee OA and this shoe style was most frequently worn, which is reflective of expert opinion. Future research is needed to confirm whether sturdy/supportive shoes are indeed optimal for managing symptoms of knee OA.

**Electronic supplementary material:**

The online version of this article (doi:10.1186/s13047-014-0043-8) contains supplementary material, which is available to authorized users.

## Background

Osteoarthritis (OA) is a major public health problem that affects nearly one in four adults worldwide [1]. The knee is commonly affected by OA, and knee OA typically results in pain, physical dysfunction and often, impairments in quality of life [2]. There is no cure for knee OA hence management is directed towards attenuating symptoms and/or minimising disease progression. A combination of pharmacological and non-pharmacological treatment is universally recommended by clinical guidelines to manage OA, and in particular, self-management strategies that are easily-administered are emphasized [2,3].

As a simple self-management strategy, international clinical guidelines recommend that health professionals advise patients with knee OA about appropriate footwear [2,4]. For instance, the European League Against Rheumatism advises footwear with thick, shock-absorbing soles and support for the arches of the foot [2], and the National Institute for Health and Care Excellence recommends footwear with shock-absorbing properties [4]. However, these recommendations are based on expert opinion alone, as there is limited evidence [5,6] on the effects of footwear on knee OA symptoms.

Despite the lack of clinical trials in relation to shock absorption and arch support, it is important to understand the type of footwear advice received by people with knee OA, as well as the common types of shoes worn, because there is the potential that footwear can influence knee OA symptoms and/or disease progression. Research has shown greater impact loads in people with knee osteoarthritis [7] and knee pain [8] compared to controls, and shoe properties such as midsole cushioning have been reported to have impact attenuating effects [9-11]. A recent uncontrolled study also demonstrated reductions in pain and symptoms with the use of cushioned insoles in a small group of people with knee OA [12]. Although there are no clinical trials proving that shoes with shock absorbing properties reduce knee OA symptoms, it is probably for these reasons that clinical guidelines advocate shoes with shock-absorbing properties.

Shoes may also influence knee OA via alterations in knee joint loading. For example, studies have shown that shoes with a greater heel height increase the external knee adduction moment (a biomechanical indicator of medial knee joint load) by 19% compared to flat shoes [13]. In contrast, lighter and more flexible shoes reduce the knee adduction moment by up to 15% compared to athletic ‘stability’ shoes [14,15]. Given that the knee adduction moment has been implicated in OA pathogenesis [16,17], as well as development of knee pain [18], and that footwear can influence this parameter, there is world-wide interest in the role of footwear in the management of knee OA. There is currently no data available about what advice people with knee OA receive regarding footwear, nor about the beliefs and wear habits of these individuals. Such information is important in order to guide the future development of novel footwear designs that could benefit knee OA, and to determine if people with knee OA are wearing footwear that may adversely influence their condition.

The objectives of this study were to describe the (a) proportion and sex differences of people with knee OA who had received footwear advice from a healthcare professional, and the nature of advice received; (b) beliefs of knee OA patients regarding footwear for their knee OA symptoms and; (c) types of footwear most frequently worn by people with knee OA.

## Methods

Ethical approval was gained from the University of Melbourne Human Research Ethics Committee (#1340549). Participants were recruited from our existing database of past and current study volunteers with knee OA, as well as through online and newspaper advertisements in metropolitan Melbourne, Australia. To be eligible, participants were required to have a clinical diagnosis of knee OA based on the American College of Rheumatism clinical criteria [19]. This was established using four survey screening questions querying whether participants: i) were aged over 50 years, ii) had knee pain on most days, iii) had morning stiffness <30 minutes and iv) had crepitus. Only people who answered yes to these questions, and consented to participate, were eligible to complete the survey.

### Survey

The survey was administered online using Survey Gizmo (www.surveygizmo.com; Widgix, LLC, Boulder, CO) and took approximately 15 minutes to complete (see Additional file 1). Survey questions were formulated based upon: (i) current and past international clinical guidelines recommending footwear for people with knee OA [2,20], (ii) scientific literature pertaining to clinical footwear trials [6] and footwear surveys [21], and (iii) consultation with medical and allied health professionals (physiotherapists and podiatrists) working with footwear and patients with knee OA. The survey was piloted by the researchers and a small number of clinicians and people with knee OA.

The survey comprised four sections. Part one contained questions about clinical characteristics including sex, age, symptom duration and pain severity. Part two queried what advice participants had received about footwear for their knee OA, and from whom. The third section ascertained participant’s beliefs about which footwear styles were beneficial for knee OA. The final section queried which footwear styles were most frequently worn by people with knee OA. Footwear style categories/properties listed within the survey were not necessarily mutually exclusive and participants were able to choose multiple options. For example, the same shoe style may have been classified by participants as “athletic shoes” and/or “sturdy/supportive shoes”, as a single shoe may share these properties. Similarly, we also included two aspects of shoe fastening separately (“buckled shoe” and “velcro-fastened shoes”) that may be found across multiple shoe styles. This was chosen to ensure that the variety of footwear styles and features advised by health professionals and described by respondents in our pilot testing was captured. Responses to parts three and four were obtained using a five-point Likert-style scale that ranged from “Strongly agree” to “Strongly disagree” or “Always” to “Never” respectively, and also included a response for “Don’t know”.

### Data analysis

Data were analysed using SPSS 21.0 (IBM, Armonk, NY, USA). Nominal and ordinal data were described as n (%), with 95% confidence intervals (CI) calculated around the proportions. All other data were reported as mean (SD) or median (interquartile range; IQR). To determine any association between receiving advice and sex, we analyzed responses from those who did and did not receive advice separately and compared data using Chi square tests. Chi square was also used to examine whether advice influenced participant’s wear habits. Where associations were found, standardized residuals were examined to identify sources of significant differences.

## Results

Of the 525 people who volunteered to participate, 390 (74%) were eligible. Of those, 186 (48%) did not complete all questions within the survey, resulting in a final sample size of 204 people (52%).

### Cohort characteristics

Table 1 lists the demographic and clinical characteristics of the sample. Sixty six males (32%) and 138 females (68%) with a mean (SD) age of 62.4 (7.7) years participated. Most (n = 146, 72%) experienced OA symptoms in both knees, and the cohort reported a median (IQR) symptom duration of 6.5 (6.0) years. Mean (SD) overall knee pain and mean (SD) knee pain on walking in the past week was 6.4 (1.4) and 6.6 (2.0) respectively.

**Table 1:** **Demographic and clinical characteristics of the sample of people with knee OA**

**Characteristics**	**n = 204**
Female, n (%)	138 (68%)
Mean (SD) age (years)	62.4 (7.7)
Affected knee, n (%)	
Left only	29 (14%)
Right only	29 (14%)
Both - left most painful	44 (22%)
Both - right most painful	64 (31%)
Both equally painful	38 (19%)
Mean (SD) duration of OA (years)	19.4 (151.6)
Mean (SD) daily pain	6.4 (1.8)
Mean (SD) walking pain	6.6 (2.0)
Current or previous treatment, n (%)	
Anti inflammatory tablets or capsules	168 (82%)
Paracetamol	179 (88%)
Topical anti inflammatory creams or gels	149 (73%)
Glucosamine or Chondroitin	159 (78%)
Oral corticosteroids	30 (15%)
Topical liniment rubs	142 (70%)
Opioid oral medication	24 (12%)
Herbal or vitamin therapies	104 (51%)
Weight loss	179 (88%)
Aerobic, strengthening or stretching exercises	165 (81%)
A walking stick, cane, walker or other object	80 (39%)
Taping of the knee cap/ knee bracing	124 (61%)
Objects to help with daily living	52 (25%)
Transcutaneous electrical nerve stimulation (TENS)	45 (22%)
Viscosupplementation	25 (12%)
Hydrotherapy	95 (47%)
Heat/cold treatment	134 (66%)
Massage therapy	68 (33%)
Acupuncture	55 (27%)
Magnet therapy	60 (29%)
Other	43 (21%)

### Advice received

Only one third of people with knee OA had received footwear advice from a health professional (n = 69, 34%), with significantly more females (n = 138, 41%) than males (n = 66, 20%) having received advice (*p* = 0.005) (Table 2). Of those that had received advice, this was most frequently received from a podiatrist (n = 47, 68%), general practitioner (n = 20, 29%) or physiotherapist (n = 20, 29%). Nearly one quarter had also received footwear advice from a non-health professional (n = 45, 22%), mainly from friends (n = 22, 49%), family (n = 19, 42%) or footwear retailers (n = 18, 40%). Significantly more females (n = 37, 27%) than males (n = 8, 12%) reported receiving footwear advice from non-health professionals (*p* = 0.029).

**Table 2:** **Number and proportion of people who received footwear advice from health professionals and non health professionals (n = 204)**

	**n (%)**
Received advice from a health professional, yes	69 (33.8%)
Health professional(s) who provided advice*	
Podiatrist	47 (68.1%)
Physiotherapist	20 (29.0%)
General practitioner	20 (29.0%)
Rheumatologist	5 (7.3%)
Surgeon	13 (18.8%)
Sports physician	5 (7.3%)
Dietician	0 (0.0%)
Psychologist	0 (0.0%)
Chiropractor	9 (13.0%)
Occupational therapist	2 (2.9%)
Exercise physiologist/exercise instructor/personal trainer	7 (10.1%)
Osteopath	2 (2.9%)
Other	3 (4.4%)
Received advice from a Non health professional, yes	45 (22.1%)
Non health professional(s) who provided advice*	
Friend	22 (48.9%)
Family member	19 (42.2%)
Internet	8 (17.8%)
Footwear retailer	18 (40.0%)
Media	2 (4.4%)
Colleague	4 (8.9%)
Other	6 (13.3%)

*Participants could respond to multiple categories hence proportions may not add up to 100%.

The shoe styles most frequently advised as good for knee OA were sturdy/supportive shoes (n = 96, 47%), shoes with in-built arch supports (n = 84, 41%) and athletic shoes/sneakers (n = 82, 40%) (Table 3). Of those styles that were advised to be bad for knee OA, the most common styles were high-heeled shoes (n = 82, 40%), thongs/flip flops (n = 58, 28%) and flexible thin soled shoes (n = 46, 23%).

**Table 3:** **Number and proportion of people who received advice regarding good or bad footwear styles for knee osteoarthritis (OA) (n = 204)**

	**Advice received**			
	**Good for knee OA**		**Bad for knee OA**	
**Footwear style**	**N**	% (95% CI)*	**N**	% (95% CI)*
**Athletic shoes/sneakers**	82	40 (34 to 47)	7	3 (1 to 6)
**Cushioned shoes**	78	38 (32 to 45)	2	1 (0 to 2)
**Sturdy/supportive shoes**	96	47 (40 to 54)	2	1 (0 to 2)
**Flexible thin soled shoes**	6	3 (1 to 5)	46	23 (17 to 28)
**Hard-soled shoes**	9	4 (2 to 7)	28	14 (9 to 18)
**Shoes with in-built arch supports**	84	41 (34 to 48)	3	1 (0 to 3)
**Lace up oxford or similar**	32	16 (11 to 21)	4	2 (0 to 4)
**Work boots**	13	6 (3 to 10)	8	4 (1 to 7)
**High heeled shoes**	2	1 (0 to 2)	82	40 (34 to 47)
**Flat shoes**	39	19 (14 to 24)	34	17 (12 to 22)
**Slip on style shoes**	10	5 (2 to 8)	38	19 (13 to 24)
**Slippers**	7	3 (1 to 6)	25	12 (8 to 17)
**Sandals**	21	10 (6 to 14)	25	12 (8 to 17)
**Clogs or ‘crocs’**	12	6 (3 to 9)	30	15 (10 to 20)
**Thongs/flip flops**	6	3 (1 to 5)	58	28 (22 to 35)
**Buckled shoes**	7	3 (1 to 6)	12	6 (3 to 9)
**Velcro-fastened shoes**	20	10 (6 to 14)	7	3 (1 to 6)
**Above ankle boots**	14	7 (3 to 10)	14	7 (3 to 10)
**Barefoot**	19	9 (5 to 13)	39	19 (14 to 24)
**Surgical/custom shoe**	14	7 (3 to 10)	2	1 (0 to 2)
**Other**	8	4 (1 to 7)	2	1 (0 to 2)

*Participants could respond to multiple categories hence proportions may not add up to 100%.

### Individual beliefs

**Figure 1:**
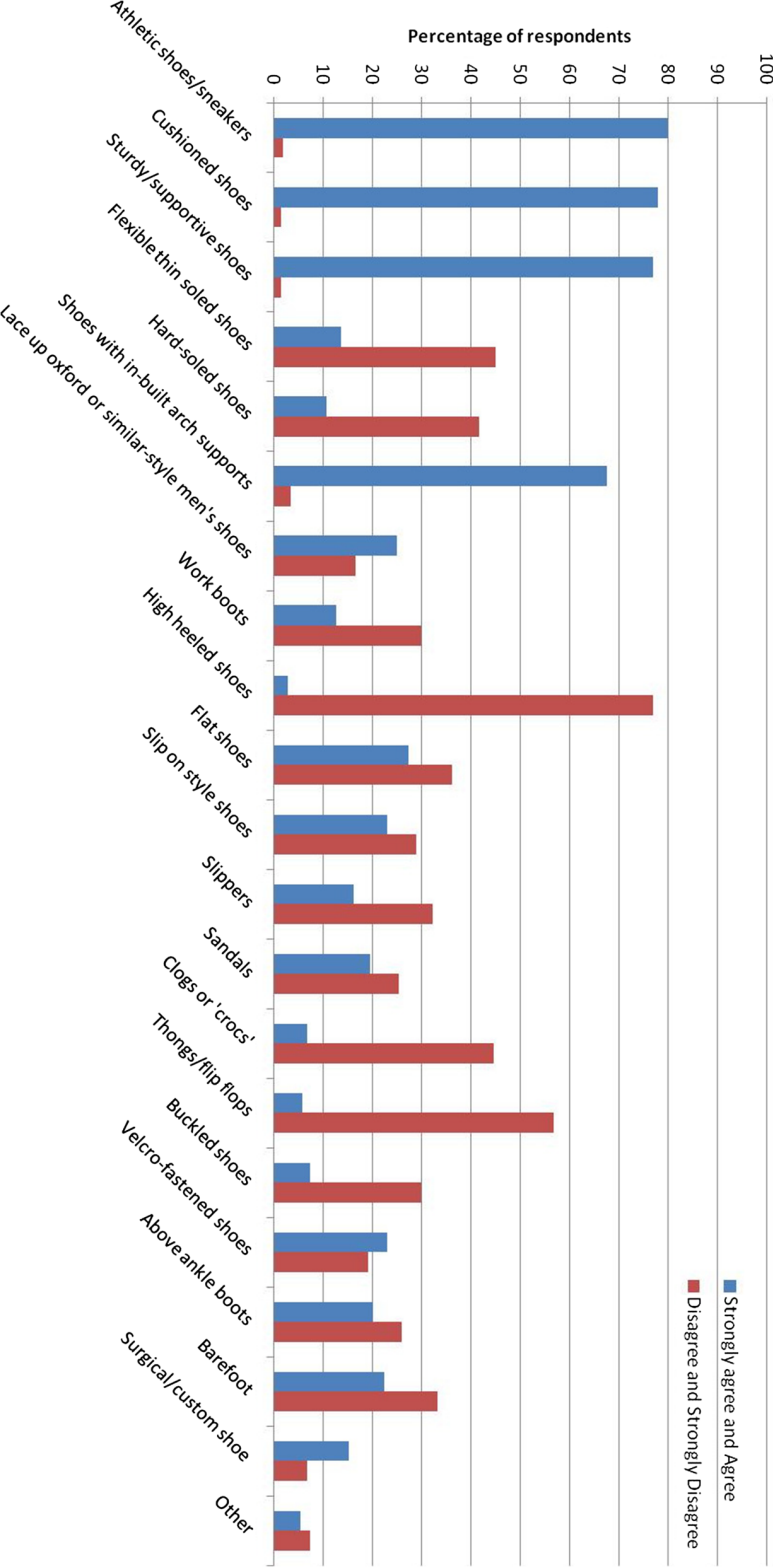
**Percentage of participants who “Strongly agreed” or “Agreed” that specific footwear styles were good for their knee osteoarthritis symptoms compared to those who “Disagreed” or “Strongly disagreed”.**

Most people agreed or strongly agreed that their knee OA symptoms were influenced by footwear (n = 150, 74%). Most believed athletic shoes (n = 163, 80%), cushioned shoes (n = 159, 78%) and sturdy/supportive shoes (n = 157, 77%) were good for knee OA (Figure 1). People with knee OA most frequently believed that high heeled shoes (n = 157, 77%), thongs/flip flops (n = 116, 57%) and flexible thin soled shoes (n = 92, 45%) were bad for knee OA.

### Shoe styles most frequently worn

**Figure 2:**
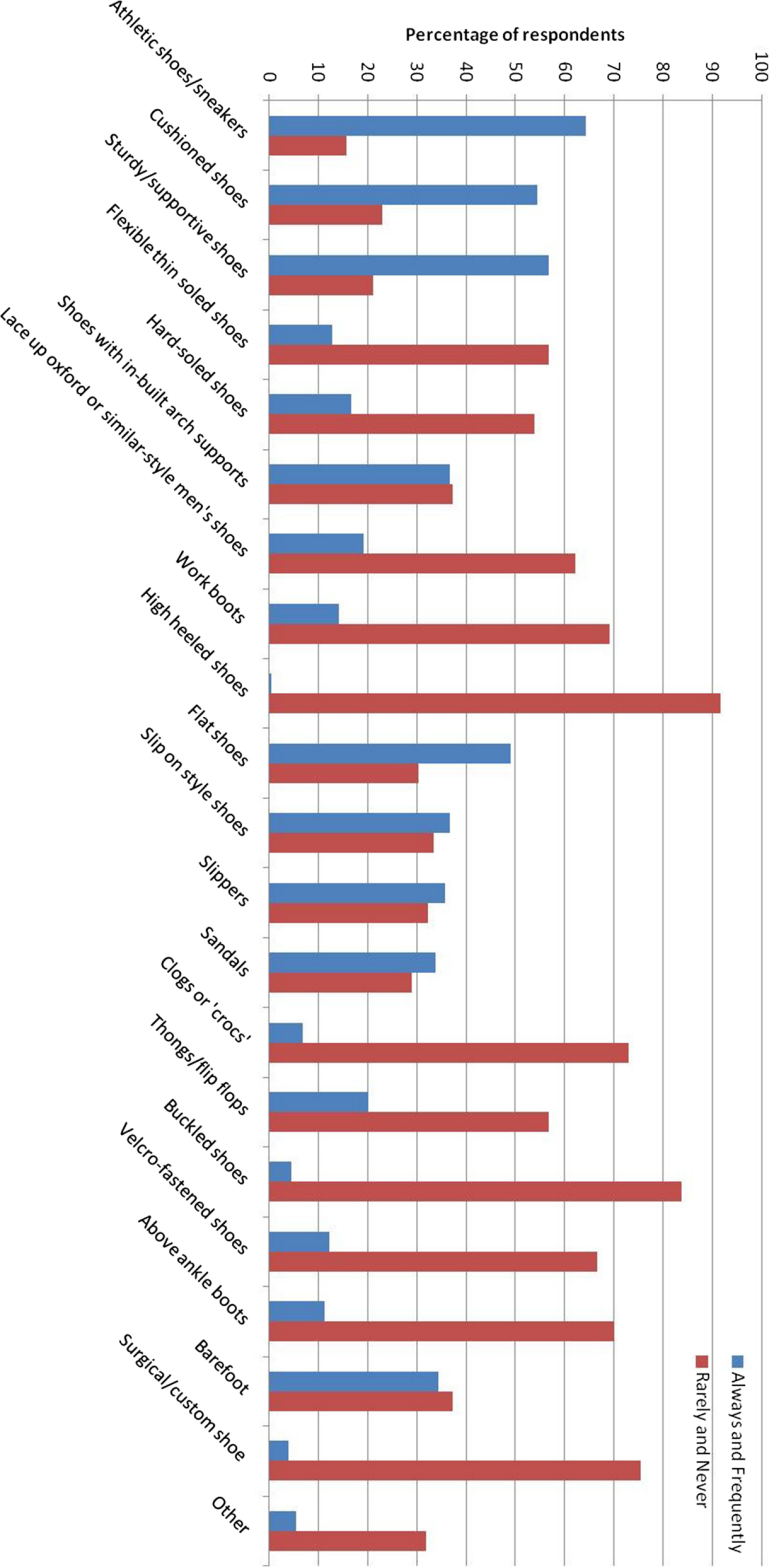
**Percentage of participants who wear specific footwear styles “Always” or “Frequently” compared to those who wear them “Rarely” and “Never”.**

Participant’s wear habits reflected their beliefs about which shoe styles are best for knee OA (Figure 2). The most commonly worn shoe style was athletic shoes (n = 131, 64%), sturdy/supportive shoes (n = 116, 57%) and cushioned shoes (n = 111, 54%). The least commonly worn shoes were high heeled shoes (n = 187, 92%), buckled shoes (n = 171, 84%) and surgical/custom shoes (n = 154, 75%). People who had received footwear advice from a health professional reported wearing hard-soled (*p* = 0.04) and work shoes (*p* = 0.02) significantly less often. No other differences in footwear habits were found between those who had and had not received advice.

## Discussion

This is the first study to investigate footwear advice, beliefs and wear habits in people with knee OA. Our results show that only one third of people surveyed had received advice from a health professional regarding footwear to manage knee OA. Health professionals overwhelmingly recommended footwear that was cushioned or promoted foot stability and/or support. Similarly, most participants believed these were amongst the best shoe choices for knee OA and were the styles worn most frequently, regardless of whether or not they had received footwear advice from a health professional.

Wearing footwear increases knee joint loading compared to barefoot walking [22], and shoe design features (such as heel height) influence the degree to which knee loading is increased [13]. Accordingly, current international clinical guidelines recommend that people with knee OA are advised to wear appropriate shoes that have no raised heel, shock-absorbing soles and support for the arches of the foot [2,4]. This recommendation is based on expert opinion alone due to the absence of research evaluating which everyday shoe styles or features are best for reducing knee OA symptoms. Although most of our participants with knee OA had not received any advice regarding footwear from a health professional, for those that had, the advice was consistent with clinical guideline recommendations. As we did not survey health professionals, it is not clear if health professionals are advising these shoe styles on the basis of their knowledge of clinical guidelines, or whether the advice they provide reflects their own individual expert opinion. We also found that more females than males had received footwear advice, although this finding likely reflects the greater health-seeking behaviour of females compared to males [23].

Participant beliefs about footwear, and their wear habits, also reflected the footwear advice received. Interestingly, footwear habits were similar between people who had and had not received footwear advice from a health professional, suggesting that people with knee OA may be influenced by footwear marketing and/or public perceptions that promote shock-absorbing stable shoe styles as optimal. Indeed, our data also demonstrate that nearly a quarter of people received advice from potentially non-qualified sources, such as family, friends and the internet, as opposed to health professionals who have training in providing health advice. The beliefs and footwear habits concerning cushioned and stable shoes in our sample are also consistent with those recently published on people with various inflammatory arthropathies [24]. However the findings from our study have direct relevance for the management of knee OA given the effects of footwear properties upon knee joint loading, a parameter known to influence knee OA pathogenesis [16,17] and knee pain [18].

Despite expert and patient opinion, it is unknown whether shoes with cushioning or stability/supportive features are actually beneficial for knee OA symptoms. In fact, biomechanical data shows that knee load is reduced in flat flexible shoes compared to athletic footwear with stability features [15,22], and uncontrolled data suggests that they can reduce pain associated with knee OA over 6 months [5]. Similarly, the addition of medial arch supports into shoes has been associated with increased knee loads [25,26], suggesting that increasing foot supination and/or restricting foot pronation may have adverse effects on loading at the knee joint. In this context, it is of interest to note that the third, fourth and sixth styles most commonly advised to be bad for knee OA symptoms by health professionals were flexible thin soled shoes, barefoot and flat shoes, respectively. Additionally, approximately half of our participants reported that they believed flexible thin soled shoes to be bad for their knee symptoms (most of the remaining participants responded “Don’t know” or “Neither agree nor disagree”), and that they rarely or never wore this shoe style. Research evaluating the effects of different shoe styles on knee OA symptoms, particularly the comparison of flat flexible shoes to stable supportive shoes, in randomized controlled trials is needed to provide evidence to inform clinical guideline recommendations regarding footwear.

Our classification of footwear styles and features may have been a limitation to the study. This was especially challenging, particularly when constructing a survey for people who may not be familiar with the technical terms describing footwear design. Thus, some of the response categories in our survey were not mutually exclusive. Athletic shoes, for example, commonly have both cushioning and support properties and therefore may be classified under each category. We felt overlap between response categories was warranted in order to capture the variety of language that may have been used by health professionals in providing footwear advice and by knee OA patients in describing their footwear beliefs and wear patterns. The large proportion of eligible participants who did not complete the survey is also a limitation which may have resulted in some bias in our data. Poor response rates [27] and incomplete responses [28] are well-recognized problems for online compared to paper-based surveys and have been attributed in part to the lack of human contact during the process [29]. In addition, it is acknowledged that aspects of our survey design, such as question layout or language complexity, may have also contributed to the high level of incomplete responses, particularly for people with poor literacy skills. Researchers have recommended including prize draws [30] or dynamic processes such as alerts or prompts in the event of incomplete answers [28], to boost the number of complete responses for online surveys. Finally, we did not query whether our participants used lateral wedges despite some evidence that laterally wedged insoles worn inside participant’s own footwear [31], and footwear that has been modified to be laterally stiff [6], can reduce knee load and pain. Future studies may consider evaluating the beliefs and wear habits of people using these devices, in addition to other orthoses/insoles, in people with knee OA.

## Conclusions

In summary, most people with knee OA have not received any specific advice about footwear for knee OA. For those that receive advice, footwear that is cushioned or promoted foot stability and/or support is most frequently recommended. People with knee OA typically believe that sturdy/supportive shoes are best for their knee symptoms, and this shoe style was most frequently worn, which is reflective of expert opinion in clinical guidelines. Future research is needed to confirm whether the shoes favoured by expert and patient opinion are indeed optimal for managing symptoms of knee OA or disease progression.

## Additional file

Additional file 1:
**Knee osteoarthritis and footwear survey.** Footwear survey used to investigate the footwear advice, beliefs and wear habits in people with knee osteoarthritis.
