# Supplementary material for: A survey of footwear advice, beliefs and wear habits in people with knee osteoarthritis
Source: J Foot Ankle Res. 2014 Oct 23;7:43. doi: 10.1186/s13047-014-0043-8 (PMC4210582; doi:10.1186/s13047-014-0043-8)
Supplement: Additional file 1: — Knee osteoarthritis and footwear survey. Footwear survey used to investigate the footwear advice, beliefs and wear habits in people with knee osteoarthritis. [file 13047_2014_43_MOESM1_ESM.pdf]

Additional file 1: A survey of footwear advice, beliefs and wear habits in people with knee osteoarthritis

THE UNIVERSITY OF MELBOURNE

KNEE OSTEOARTHRITIS AND FOOTWEAR SURVEY

Part 1 - About yourself

1. **What is your gender?** Male ☐ Female ☐

2. **What is your date of birth?** ☐☐ ☐☐ 1 9 ☐☐
   Date Month Year

3. **In which knee is your osteoarthritis:**

   Left ☐ Right ☐ Both – left worse ☐
   Both – right worse ☐ Both – left and right equal ☐

4. **How long have you experienced knee osteoarthritis symptoms?** ☐☐ ☐☐
   Years Months

5. **Place a tick on the following scale to show the <u>average amount of pain felt over the PAST WEEK</u> in your osteoarthritic knee (if you have symptoms in both knees please only indicate the pain in your most painful knee):**

☐	☐	☐	☐	☐	☐	☐	☐	☐	☐	☐
0	1	2	3	4	5	6	7	8	9	10
no pain										worst pain possible

6. Place a tick on the following scale to show the <u>average amount of pain felt over the PAST WEEK</u> in your osteoarthritic knee <u>when you are walking</u> (if you have symptoms in both knees please only indicate the pain in your most painful knee):

☐	☐	☐	☐	☐	☐	☐	☐	☐	☐	☐
0	1	2	3	4	5	6	7	8	9	10
no pain										worst pain possible

Part 2 – Advice you have been given about footwear for your knee osteoarthritis

7. Have you ever received advice about what footwear or footwear features (e.g. high heels) you should or should not wear for your knee osteoarthritis from a <u>health professional?</u>

Yes ☐

No ☐

If no, go to question 9

8. If you have received advice about footwear for your knee osteoarthritis, which <u>health professional(s)</u> provided this advice (select all that apply):

Podiatrist ☐	Physiotherapist ☐	General practitioner ☐
Rheumatologist ☐	Surgeon ☐	Sports physician ☐
Occupational therapist ☐	Exercise physiologist / exercise instructor / personal trainer ☐	Chiropractor ☐
Osteopath ☐		

Other (please describe): ____________________

9. Have you ever received advice about what footwear or footwear features you should or should not wear for your knee osteoarthritis from a <u>NON-health professional</u> (e.g. a friend)?

Yes ☐

No ☐

If no, go to question 11

10. If you have received advice about footwear for your knee osteoarthritis from <u>a NON-health professional</u>, who provided this advice (select all that apply):

Friend ☐	Family member ☐	Internet ☐
Footwear retailer ☐	Media ☐	Colleague ☐
Other (please describe):		

11. Have you ever been told that any of the following footwear choices will be <u>good or bad</u> for your knee osteoarthritis (you may select more than one option):

	Bad	Good	No advice given
Athletic shoes/sneakers	☐	☐	☐
Cushioned shoes	☐	☐	☐
Sturdy/supportive shoes	☐	☐	☐
Flexible thin soled shoes	☐	☐	☐
Hard-soled shoes	☐	☐	☐
Shoes with in-built arch supports	☐	☐	☐
Lace up oxford or similar-style men's shoes	☐	☐	☐
Work boots (lace up or slip on)	☐	☐	☐

High heeled shoes	☐	☐	☐
Flat shoes	☐	☐	☐
Slip on style shoes	☐	☐	☐
Slippers	☐	☐	☐
Sandals	☐	☐	☐
Clogs or 'crocs'	☐	☐	☐
Thongs/flip flops	☐	☐	☐
Buckled shoes	☐	☐	☐
Velcro-fastened shoes	☐	☐	☐
Above ankle boots	☐	☐	☐
Barefoot	☐	☐	☐
Surgical/custom shoe (please describe):	☐	☐	☐
Other (please describe):	☐	☐	☐

Part 3 – Your own beliefs about footwear for knee osteoarthritis

12. How much do you feel that your <u>knee osteoarthritis symptoms</u> are influenced by wearing different types of footwear:

Strongly agree	Agree	Neither agree or disagree	Disagree	Strongly disagree	Don't know
☐	☐	☐	☐	☐	☐

13. **How much do you feel that wearing each of the different types of footwear below could be <u>good for your knee osteoarthritis symptoms</u> (please indicate one response for each type):**

	Strongly agree	Agree	Neither agree or disagree	Disagree	Strongly disagree	Don't know
Athletic shoes/sneakers	☐ 0	☐ 1	☐ 2	☐ 3	☐ 4	☐ 5
Cushioned shoes	☐ 0	☐ 1	☐ 2	☐ 3	☐ 4	☐ 5
Sturdy/supportive shoes	☐ 0	☐ 1	☐ 2	☐ 3	☐ 4	☐ 5
Flexible thin soled shoes	☐ 0	☐ 1	☐ 2	☐ 3	☐ 4	☐ 5
Hard-soled shoes	☐ 0	☐ 1	☐ 2	☐ 3	☐ 4	☐ 5
Shoes with in-built arch supports	☐ 0	☐ 1	☐ 2	☐ 3	☐ 4	☐ 5
Lace up oxford or similar-style men's shoes	☐ 0	☐ 1	☐ 2	☐ 3	☐ 4	☐ 5
Work boots (lace up or slip on)	☐ 0	☐ 1	☐ 2	☐ 3	☐ 4	☐ 5
High heeled shoes	☐ 0	☐ 1	☐ 2	☐ 3	☐ 4	☐ 5
Flat shoes	☐ 0	☐ 1	☐ 2	☐ 3	☐ 4	☐ 5

Slip on style shoes	☐ 0	☐ 1	☐ 2	☐ 3	☐ 4	☐ 5
Slippers	☐ 0	☐ 1	☐ 2	☐ 3	☐ 4	☐ 5
Sandals	☐ 0	☐ 1	☐ 2	☐ 3	☐ 4	☐ 5
Clogs or ‘crocs’	☐ 0	☐ 1	☐ 2	☐ 3	☐ 4	☐ 5
Thongs/flip flops	☐ 0	☐ 1	☐ 2	☐ 3	☐ 4	☐ 5
Buckled shoes	☐ 0	☐ 1	☐ 2	☐ 3	☐ 4	☐ 5
Velcro-fastened shoes	☐ 0	☐ 1	☐ 2	☐ 3	☐ 4	☐ 5
Above ankle boots	☐ 0	☐ 1	☐ 2	☐ 3	☐ 4	☐ 5
Barefoot	☐ 0	☐ 1	☐ 2	☐ 3	☐ 4	☐ 5

Special shoe (please describe):						
	☐ 0	☐ 1	☐ 2	☐ 3	☐ 4	☐ 5
Other (please describe):						
	☐ 0	☐ 1	☐ 2	☐ 3	☐ 4	☐ 5

Part 4 – Your shoe wear habits

14. Please rate how often you wear the following footwear choices (please chose one response for <u>each</u> type):

	Always	Frequently	Occasionally	Rarely	Never	Don't know
Athletic shoes/sneakers	☐	☐	☐	☐	☐	☐
Cushioned shoes	☐	☐	☐	☐	☐	☐
Sturdy/supportive shoes	☐	☐	☐	☐	☐	☐
Flexible thin soled shoes	☐	☐	☐	☐	☐	☐
Hard-soled shoes	☐	☐	☐	☐	☐	☐
Shoes with in-built arch supports	☐	☐	☐	☐	☐	☐

Lace up oxford or similar-style men’s shoes	☐	☐	☐	☐	☐	☐
Work boots (lace up or slip on)	☐	☐	☐	☐	☐	☐
High heeled shoes	☐	☐	☐	☐	☐	☐
Flat shoes	☐	☐	☐	☐	☐	☐
Slip on style shoes	☐	☐	☐	☐	☐	☐
Slippers	☐	☐	☐	☐	☐	☐
Sandals	☐	☐	☐	☐	☐	☐
Clogs or ‘crocs’	☐	☐	☐	☐	☐	☐
Thongs/flip flops	☐	☐	☐	☐	☐	☐
Buckled shoes	☐	☐	☐	☐	☐	☐
Velcro-fastened shoes	☐	☐	☐	☐	☐	☐
Above ankle boots	☐	☐	☐	☐	☐	☐
Barefoot	☐	☐	☐	☐	☐	☐
Surgical/custom shoe (please describe):						
	☐	☐	☐	☐	☐	☐
Other (please describe):						
	☐	☐	☐	☐	☐	☐
